# Letter to the editor: Epidemiology of heart failure hospitalization in patients with stable atherothrombotic disease: The contribution of previous heart failure to heart failure hospitalization may be overestimated

**DOI:** 10.1002/clc.23973

**Published:** 2023-01-19

**Authors:** Kaibo Mei, Qin Ling, Yan Gao, Xiao Liu, Zhiwei Yan

**Affiliations:** ^1^ Department of Anesthesiology The People's Hospital of Shangrao Jiangxi China; ^2^ Endocrine Department The Second Affiliated Hospital of Nanchang University Jiangxi China; ^3^ Department of Sports Rehabilitation, College of Human Kinesiology Shenyang Sport University Shenyang Liaoning China; ^4^ Department of Cardiology Sun Yat‐Sen Memorial Hospital of Sun Yat‐Sen University Guangzhou Guangdong China

To the Editor,

We have read with great interest the article by Freedman et al.,^1^ highlighting that the risk of heart failure hospitalization (HHF) is higher in stable atherosclerosis patients with previous heart failure, type 2 diabetes mellitus, and polyvascular disease. After careful reading, we would like to present some opinions from different perspectives.

Freedman and coworkers reported that the risk of HHF in stable atherosclerotic cardiovascular disease (ASCVD) patients was eightfold higher in those with a history of heart failure (HF) (odds ratio [OR] = 8.31, 95% confidence interval [CI]: 6.56–10.54). We supposed that the impact of the history of F on the risk of HHF in stable ASCVD patients may be overestimated For example, Malik et al.^2^ showed that prior HF hospitalization was associated with 32% and 48% increased heart failure readmission in heart failure with preserved (HFpEF) and reduced ejection fraction (HFrEF), respectively (hazard ratio = 1.32, 95% CI: 1.23–1.41 and 1.48, 95% CI: 1.37–1.61). Theoretically speaking, the contribution of previous HF to HHF in ASCVD is relatively lower than that in patients with HF. Several residual confounding factors may be responsible. For example, atrial fibrillation (AF) is quite common in patients with HF and is associated with a higher risk of hospitalization.^3^ Furthermore, AF and HF share common risk factors and mutually contribute to poor prognosis, which is called the “Vicious Twins” hypothesis proposed by Kotecha et al.^4^ As a result, it is important to proactively adjust for baseline AF in outcomes for patients with HF.

It should also be noted that Freedman et al. concluded that antiplatelet therapy would not reduce the risk of HHF in ASCVD patients, and they attributed the cause to insufficient follow‐up time or to events other than myocardial infarction. However, they did not distinguish between the first HF hospitalization and the second HF hospitalization. The first HF hospitalization was an indicator of HF incidence, and the second or readmission was cardiovascular events in HF patients. Mixed HF hospitalization may introduce a proportion of patients with HF. The 2021 European Society of Cardiology Guidelines for the diagnosis and treatment of acute and chronic heart failure^5^ did not recommend antiplatelet therapy in patients with HF coexisting with coronary artery disease, suggesting that antiplatelet therapy may have no benefit for these patients. Therefore, mixed HF patients may have an effect on the association between antiplatelet therapy and adverse outcomes in ASCVD patients, which should be further discussed.

## AUTHOR CONTRIBUTIONS

Under the directions of Xiao Liu and Ziwen Yan, Kaibo Mei and Qin Ling drafted the first version of the manuscript. Dr Liu revised and prepared the final version of the manuscript.

## CONFLICT OF INTEREST

The authors declare no conflict of interest.
